# The future of work in shaping the employment inclusion of young adults with disabilities: a qualitative study

**DOI:** 10.1108/EDI-06-2022-0154

**Published:** 2023-05-09

**Authors:** Arif Jetha, Ali Shamaee, Emile Tompa, Peter Smith, Ute Bültmann, Silvia Bonaccio, Lori B. Tucker, Cameron Norman, Cristina G. Banks, Monique A.M. Gignac

**Affiliations:** Institute for Work and Health, Toronto, Canada; Dalla Lana School of Public Health, University of Toronto, Toronto, Canada; Institute for Work and Health, Toronto, Canada; Institute for Work and Health, Toronto, Canada; Department of Economics, McMaster University, Hamilton, Canada; Dalla Lana School of Public Health, University of Toronto, Toronto, Canada; Institute for Work and Health, Toronto, Canada; Dalla Lana School of Public Health, University of Toronto, Toronto, Canada; University Medical Centre Groningen, Groningen, The Netherlands; Telfer School of Management, University of Ottawa, Ottawa, Canada; BC Children’s Hospital, Vancouver, Canada; Faculty of Medicine, University of British Columbia, Vancouver, Canada; Cense LTD, Toronto, Canada; University of California Berkeley, Berkeley, California, USA, and; Institute for Work and Health, Toronto, Canada; Dalla Lana School of Public Health, University of Toronto, Toronto, Canada

**Keywords:** Future of work, Disability, Young adults, Changing nature of work, Digital transformation, Precarious work, Automation

## Abstract

**Purpose –:**

The world of work is changing and creating challenges and opportunities for the employment inclusion of young people with disabilities. In this article, the perceptions held by young adults with disabilities regarding participation in the future of work are examined.

**Design/methodology/approach –:**

One-on-one interviews were conducted with Canadian young adults (ages 18–36 years) living with a disability. Participants were asked about their thoughts regarding the impact of the changing nature of work on their labor market involvement and career aspirations. A thematic analysis was performed to identify and examine emergent salient themes.

**Findings –:**

In total, 22 young adults were interviewed; over half held secure employment. Career aspirations and work-related decisions were primarily shaped by a participant’s health needs. The future of work was seen as a more proximal determinant to employment. Digital technologies were expected to impact working conditions and create barriers and facilitators to employment. Participants who indicated being securely employed held positive expectations regarding the impact of digital technology on their work. Participants working precariously held negative appraisals regarding the impact of digital technologies on employment opportunities. The role of technological and soft skills was critical to participating in a labor market reliant on advanced technology. Participants reported barriers to developing job skills related to their disability and their work arrangements.

**Originality/value –:**

This research highlights the importance of considering changes in the future of work, especially the digital transformation of the economy, in the design of initiatives which promote the employment inclusion of young adults with disabilities. Despite the significance of the changing nature of work, supporting health needs and encouraging access to secure work arrangements also remain paramount.

## Background

The future of work, characterized by large-scale interrelated technological, sociopolitical and environmental trends, is expected to transform all aspects of the working world and contribute to employment and health inequities (World Economic Forum, 2021; Russek et al., 2021; Schneider et al., 2017). Few studies have examined how the future of work creates challenges and opportunities to sustained and inclusive employment for vulnerable groups of workers, such as persons living with disabilities. Our study focuses on young adults with disabilities at the early phase of their career who face barriers to entering and advancing within the working world and indicate requiring workplace and policy-level supports for sustained employment (Jetha et al., 2019). The future of work has the potential to dramatically alter the working context to which young adults with disabilities are exposed and affect early and long-term labor market inclusion.

The future of work is a dynamic topic that encompasses a diverse set of forces expected to disrupt every industry, change work contexts and impact occupations and job availability (Schneider et al., 2017; Organisation for Economic Co-Operation and Development, 2020; Russek et al., 2021; World Economic Forum, 2021). A prominent feature of the future of work is the digital transformation of the economy which refers to the advancement and application of diverse digital technologies. The digital transformation of the economy is occurring at a faster pace when compared to past periods of technological adoption and can contribute to the automation of work, changing the job task composition of occupations and an evolving employer demand for workers with specific technical and soft job skills (Bick et al., 2020; Manyika et al., January 2017; Acemoglu and Restrepo, 2020; Baldwin, 2019; Jetha et al., 2021). The digital transformation of the economy can also contribute to an erosion of standard employment opportunities and a rise in contingent employment (e.g. digital platform work and gig work). The future of work can also be related to social (e.g. generational composition of the workforce), political (e.g. populist sentiments) and environmental changes (e.g. climate change) that have significant implications for workers (Jetha et al., 2021, World Economic Forum, 2021; Russek et al., 2021). Of concern, workers who have previously faced disadvantage within the labor market, including those employed in precarious and lower-skilled jobs, may be more likely to be adversely affected by the digital transformation of the economy and other drivers of change in the future of work (Acemoglu and Restrepo, 2018; Lamb and Doyle, 2016; Jetha et al., 2021). Accordingly, the future of work may play a significant role in determining inclusion within the workplace and the socioeconomic position of workers (Burgard and Lin, 2013; Benach et al., 2000).

Young adults with disabilities represent one group who have been traditionally disadvantaged within the labor market and may be particularly affected by changes in the future of work. In Canada, where the current study was conducted, close to two million youth and young adults live with a disability (Morris et al., 2018). Studies indicate that a disability in young adulthood can impact entry and advancement in the working world and shape the aspirations one has towards their career (Morris et al., 2018). Existing Canadian population-level data show that the employment rate of youth (18–24 years, 32%) and young adults (25–35 years, 54%) living with a disability is significantly lower than their counterparts without a disability (52 and 82%, respectively) (Morris et al., 2018). Young adults with disabilities who are employed are more likely to report being excluded from high quality employment (e.g. secure work arrangements and safe work) and career advancement opportunities and are more likely to report precarious employment, fewer work hours, lower income, lost productivity and greater barriers to career advancement than their non-disabled peers (Morris et al., 2018; Jetha et al., 2019; Mann and Honeycutt, 2014; Mann and Wittenburg, 2015). Of concern, research indicates that employment challenges faced by young adults living with disabilities at the early career phase can have an economic scarring effect that impacts employment opportunities across the working life course.

Widely used biopsychosocial models of disability, such as the World Health Organization’s International Classification of Functioning Disability and Health, highlight that disability stems from the interaction between health (e.g. disability severity), personal (e.g. age, sex/gender, educational attainment) and contextual factors (e.g. working condition, employment arrangements, availability of job accommodations) which can create barriers or facilitators to participation in different domains of life including employment (Üstün et al., 2003). Within the context of biopsychosocial models of disability, the future of work can dramatically change the working context to which persons living with disabilities are exposed and create novel barriers and facilitators to employment participation.

Limited research has examined how the future of work will impact the employment inclusion of young adults with a disability. A small number of studies suggest that young adults with disabilities are overrepresented in working contexts characterized by low-skill, low-pay and physically demanding work which may be adversely affected by the digital transformation of the economy and other labor market disruptions (Maroto and Pettinicchio, 2014; Tompa et al., 2020). Changes in the future of work have the potential to exacerbate existing vulnerabilities faced by young adults living with disabilities and contribute to new challenges. At the same time, emerging occupations and an employer demand for workers with evolving job skills represent critical opportunities for the inclusion of young adults with disabilities in the labor market.

Currently, there is a lack of evidence to inform the design of strategies that are utilized by employers, disability service providers, policymakers or other professionals which can support the employment participation of young adults with disabilities and are also resilient to the changes in the future of work. Furthermore, research on the future of work is required to advance a conceptual understanding of the implications of evolving work context factors impacting barriers and facilitators to employment participation for young adults with disabilities. Our study addresses two overarching research objectives, which include to:

1. Examine how young adults with disabilities view their long-term careers and the role of the future of work in shaping their employment experiences.
2. Explore how changes in the future of work impact access to the resources and supports required by young people living with disabilities to sustain employment.

## Methods

We conducted one-on-one interviews with Canadian young adults (ages 18–36 years) with a self-reported disability. Interviews represented an important approach to obtain a deeper understanding of the current employment experiences and perceptions held by participants regarding the future of work. Interviews also allowed the research team to investigate key themes that were raised during data collection and allowed participants to elaborate on their unique lived experience. To capture breadth and depth in thoughts regarding the future of work, we recruited participants who differed according to gender, disability type and employment. Interviewees were identified through an existing cohort of young adults with disabilities who participated in a previous study led by our team and had consented to being contacted for research (Jetha et al., 2019). Participants recruited for this study were currently employed, had previous employment experience, or were planning to enter the workforce. Additionally, participants recruited for this study were required to be comfortable talking about their employment experiences and did not face restrictions to informed consent.

Email invitations and a study background were sent to potential participants. Interested participants were asked to contact the research team to confirm eligibility, provide informed consent and schedule an interview. The interview process began in February 2020, and a rolling recruitment strategy was taken to address emergent themes. Our study was conducted during the early stages of the COVID-19 pandemic. Additional interviews were conducted to capture emerging themes related to the impact of COVID-19 on working experiences. Participant recruitment occurred until no new themes emerged (Varpio et al., 2017). Participants received a $25 honorarium for their involvement. To protect confidentiality, participants were only asked to share details regarding their work experiences that they would be willing to share publicly. Personal identifying information was removed from interview transcripts and all participants were assigned a unique identifying number. All data was stored in an encrypted digital folder that was only accessible to members of the research team. Study procedures were reviewed by the corresponding author’s institutional research ethics board.

### Interview questions

Interviews were semi-structured and conducted over the phone. Interviews began with an introduction to the concept of the future of work to provide the interviewee with insights necessary to engage with the topic. Interview questions asked about career aspirations and how a disability could impact meeting career goals. Participants were probed on their thoughts and perceptions regarding technological, sociopolitical, economic and environmental dimensions of the future of work and how these could affect their employment experiences. All interviews were recorded and transcribed verbatim and checked for accuracy.

### Data analysis

Our study took a constructivist epistemological perspective (Miles et al., 2018; Denzin and Lincoln, 2011). While we utilized theory and literature on the future of work and disability to guide the development of our interview guide, we followed a thematic analysis approach to inductively identify and examine emergent themes, build a relationship between themes, and construct an understanding of the implications of the future of work (Miles et al., 2018; Braun and Clarke, 2012). Transcripts were reviewed by three members of the research team, and an initial codebook was collaboratively developed and revised through several iterative conversations. Utilizing the codebook, two transcripts were coded by four investigators to ensure dependability. The codebook was refined through additional discussions with the investigative team and applied to the remaining transcripts. A primary and secondary coder conducted comprehensive line-by-line coding of transcripts. Once coding was completed, prominent themes or patterns in the data were inductively extracted and specific codes nested within each theme were identified. Codes and themes emerging from the data were discussed in analysis meetings with some members of the investigative team at which time any inconsistencies were resolved by consensus. Our analysis leveraged expertise from a multidisciplinary research team with expertise in qualitative research methods. NVivo was utilized to facilitate coding and thematic analysis (QSR International Pty Ltd, 2016).

## Results

### Sample description

We interviewed 22 participants (Table 1). Close to two-thirds of participants (64%) identified as women and one participant identified as non-binary. Over half worked full-time (55%; ≥ 30 h/week), 27% worked part-time hours (<30 h/week), and 18% reported not working or being furloughed because of the COVID-19 pandemic. Fifty five percent indicated a physical disability, and 45% reported a mental health or cognitive disability.

### Summary of themes

Emerging from the data were three broad thematic categories which contained multiple subthemes. The first thematic category (Thematic Category A) was related to career aspirations and work-related decisions. When asked about the future of work, young adults with disabilities who participated in our study described that health and disability was the primary determinant of career decisions rather than exogenous changes to the nature and availability of work. Most participants indicated that their career aspirations and work-related decisions were primarily shaped by their support needs. Within Thematic Category A, the future of work was seen as a more proximal determinant to participants’ ability to find and sustain employment. The second thematic category (Thematic Category B) was related to the role of digital technologies in transforming working conditions for young adults with disabilities. This theme contained parallels to the first theme related to long-term changes and employment sustainability, availability of workplace supports and additional skills training and the job security (or lack thereof) afforded by technology. The third category (Thematic Category C) related to job skills needed in an economy undergoing a digital transformation and the barriers to obtaining job skills.

### Thematic Category A: career aspirations and work decisions shaped by accommodation needs and not future of work changes

#### Theme A1: Disability creates apprehension to think about the future of work.

Discussions on the future of work centered on whether a participant felt like they could meet their career aspirations as well as their longer-term employment expectations. Despite including multiple probes in our interview guide regarding changes in the future of work, there was a general apprehension among participants to think about their working life too far ahead. Most participants anticipated that their health would worsen, or their disability would contribute to a greater number of activity limitations that would restrict employment in ways that could make meeting their career goals more challenging. Participants reported that they were less likely to feel that different aspects of the future of work could impact their long-term career. For instance, one participant who lived with a mental health disability and was employed in multiple part-time jobs at the time of the interview reflected on perceptions of the future, noting that:

It’s difficult for me to think about the future because I used to make plans and then life would go in very unexpected ways, so I just stopped. I’m trying [to] re-grow that part of myself, the part that can sort of make plans and try and think about the future.

- Participant ID#164

Participants indicated taking a shorter-term perspective towards their career and prioritizing the management of their disability when making employment decisions. These decisions were often directly tied to their disability and health characteristics and their requirement to find employment that was well-suited to their support needs. One participant with a mental health disability who was not working highlighted their goal of finding employment that could support a disability with fluctuating activity limitations:

I know that my health can fluctuate a lot in a year, it goes from feeling fine to not several times over a year. Because it’s unpredictable it can affect everything. It can affect how well I can look for a job and how well I can perform at a job. It could have a big impact. I also think that the wrong job could, if I was in a job that was too high stress or they’re not accommodating enough, it could make mental health worse just on its own.

- Participant ID#128

The requirement to find supportive work was exacerbated for those with a progressively worsening disability. One participant living with a physical disability who held a full-time permanent job with a supportive employer at the time of the interview expected that their health condition would become more severe. The participant indicated hesitancy about leaving their current job to pursue longer-term career aspirations even if it meant that their job could be disrupted by changes in the future of work:

… my disability is one that progresses with time. I will get weaker as time goes on. That obviously is something I must think about [when making career-related decisions]. Will my job continue to be flexible in that way? Like I said, I think it would be very hard for me to want to leave this [job] and go to another job not knowing how they [employer] would treat this type of situation.

-Participant ID#117

Participants repeatedly described that their career aspirations included seeking a job that could support their disability. Most participants, especially those with a disability that had more complex treatment and self-management requirements, emphasized the importance of obtaining a job with security and where formal job accommodations were readily available. Participants also talked about the importance of employment within certain sectors (e.g. banking, public service) where accessible working conditions were commonplace. For example, one participant with a physical disability who was employed in a permanent fulltime job at an academic institution described that their requirement for employer-provided drug plans shaped their career decisions:

Yeah, I can’t [think about leaving current employment]… If I ever were to apply to a job at a different corporation or university, I would have to first ask, do their benefits package cover the [drug] for the amount that I have, and covers not things that aren’t pre-existing, that existed after the fact? … It makes it a little bit more pointless to even apply to jobs if I know that’s the case, so I’ve more or less have had to resign myself to continue to work at my institution as it stands.

–Participant ID#102

#### Theme A2: importance of job security in career aspirations with a disability.

To sustain employment participation with a disability, young adult participants in our study emphasized the importance of selecting and retaining a secure job with workplace supports. At the same time, obtaining secure employment was seen by participants as being challenging within the context of current and future labor market conditions. Participants frequently described that the availability of secure employment arrangements was decreasing (e.g. full-time permanent work) and contingent arrangements (e.g. shorter-term contracts, seasonal work, gig work, freelancing and microtasks) were more commonly available. In conversations on the future of work, participants expected that the increasing likelihood of working in a contingent arrangement stemmed from the growing adoption of digital technologies that automated job tasks, or an increasing number of digital applications being used by companies that facilitated contracting of short-term jobs or gigs. One participant described working as a customer service representative where job tasks were being automated and there was competition for a limited number of shifts. The same participant described the desire for greater job security and scheduling predictability:

Things gradually improve and then what’s good one week it can revert to something bad the following week. We were getting more projects overall at the [company name], like my company, who are third party for [company name]. But now one of the projects is going to end. So, then all those people are basically out of work. So I’m going to be competing against them for the finite number of shifts that are available. So, me getting four shifts a week it’s not likely anymore. I may only get two shifts a week and then that affects your income. And then at that point you’re like why I should stick it out with this job. Where can I go to another job and get five days a week guaranteed every week?

– Participant ID#121

Participants felt that having a disability meant that they were more likely to be excluded from secure employment arrangements and forced to work contingently when compared to their peers not living with a disability. It is important to highlight that some participants acknowledged that contingent work arrangements could offer benefits to people with disabilities including an opportunity to obtain work experience or to acquire scheduling flexibility. However, participants most frequently described negative consequences of contingent work such as being excluded from legislative protections, not having access to workplace supports or feeling that their employment was precarious. One participant in a full-time permanent job with a physical disability reflected on the implications of insecure gig work for people with disabilities:

A lot of times, the gig economy is seen as a good thing, that maybe it will include people with disabilities furthermore. I’m sure that’s about the reality too. But I think the gig economy could be quite dangerous for people with disabilities, the inclusion of people with disabilities specifically, partly because right now accommodations are enshrined in [employment legislation]. So, when you move into a gig economy, you are no longer an employee, you are a contractor.

- Participant ID#101

In sum, the first overarching theme that emerged from the data indicated that participant’s career aspirations were less likely to be shaped by changes in the future of work. Work-related decisions were primarily driven by the need to support their health and constrained by access to secure employment arrangements. As describe in the Thematic Category B, despite a hesitance to think too far into the future, participants expected that technological changes would affect employment conditions and the way they performed their jobs.

### Thematic Category B: digital technologies could transform working conditions and change job skill requirements

The digital transformation of the economy was considered by participants as the most predominant factor that could change working conditions in the future. In Thematic Category B, participants described examples of technology that they encountered within their work that affected their employment, such as artificial intelligent (AI) systems, cloud computing, Internet of Things devices and virtual reality. Advancements in these digital technologies were described by participants as having both opportunities and barriers for people with disabilities.

#### Theme B1: securely employed participants were more likely to highlight digital technology benefits.

Some noted that certain digital technologies could be used as a tool to provide greater flexibility to work schedules and job task performance, support work-from-home arrangements and reduce physical job demands. Interestingly, participants who worked securely and in white-collar jobs were more likely to hold positive evaluations regarding the impact of digital technology compared to those in contingent employment. For example, one participant who worked full-time described the benefits of digital technologies in enabling work-from-home arrangements to improve employment accessibility for people with disabilities:

… if there’s more advances in technology where people can have the same software they’re using at work and being able to use that at home, I think it will give, especially for people with disabilities, more opportunities to be able to have a job and keep a job. Like, with our company right now, they’re looking at options for cloud software for us to use from our computers … I think facilitating work at home, maybe enable access to other spaces that wouldn’t normally be accessible to people otherwise …

- Participant ID#173

The role of digital technologies was often discussed within the context of the COVID-19 pandemic. Those securely employed noted that their workplaces increased the provision of digital platforms to facilitate work-from-home arrangements in response to COVID-19. The opportunity to perform job duties from home was seen as an important change to working conditions that enabled people with disabilities to manage their disability. At the same time, participants indicated frustration regarding being denied similar accommodations in the past:

I think that’s the number one important thing, to have good technology for all these companies, to be able to accommodate all of their employees. For example, during that time when I was looking for a job and I applied to places and I would ask them about options for working from home, a lot of them would say, oh, no, we don’t do that. But now, with this virus that is happening, almost everyone is working from home, and I know a lot of those companies have employees that are working from home right now. I’m like, oh, was it just because of my disability, that you didn’t want to hire me, or you just weren’t thinking ahead in that kind of way at that time? It’s something that I was thinking about because I feel like it’s very easy for a lot of companies to do this. I think technology is very important.

– Participant ID#117

#### Theme B2: digital technology is considered disadvantageous for those working in contingent employment.

Digital technology was not seen as beneficial to all participants. Those employed in contingent work or not employed reported more concerns about the application of digital technology to their ability to sustain long-term employment. Participants who indicated that their employment was precarious described automated devices (e.g. digital kiosks) or AI-enabled systems (e.g. chatbot service representatives) as examples of digital technologies that reduced the availability of work shifts and the number of job tasks for which they were responsible and also increased competition for a limited number of employment positions. Those in contingent work arrangements felt that advancements in the development and application of different digital technologies within their workplaces would negatively impact their employment and contribute to greater precarity or job displacement all together. For example, one participant working precariously in the service industry highlighted the perceived risk of work being automated:

I don’t think technology would be a good thing in my job because … customers could interface with a computer screen, and it tells them everything they want to know. What use are us to be there? We’re dispensing information. The more technologically advanced things get it’s the less likely they’ll [employer] need us. Even the [fare] collectors, there’s no more collectors in the [company] anymore because they’ve been phased out because of [digital payment systems]. Technology is a good thing but then … it’s a bad thing too because you need that human connection sometimes that’s kind of missing when you just deal with a robot.

– Participant ID#121

The intersection between digital technologies and contingent employment resulted in participants emphasizing the importance of employer- or policy-level supports to protect people with disabilities. One participant noted that in light of increasing employer investments in the design and application of digital technologies, there should be workplace policies and managerial practices that provide job security and offer support to workers with disabilities:

I think that technology can only do so much if those technological advances are not paired with structural and institutional attitudes of accessibility and … When I look at technological advances, they seem to mostly prioritize an ethic of productivity. And so, when it comes to disabled workers, technological advancement help business and help employers because now disabled workers can be just as productive as a non-disabled worker. And absolutely when disabled people, we live precarious lives and we don’t know what our future will look like in terms of our income, and our access to social benefits are very limited in this province. We want to be working and we want to feel secure in that work.

– Participant ID#069

#### Theme B3: workers with disabilities can offer unique contributions to an increasingly automated and digital working world.

The increasing reliance on machines to automate work tasks was described as creating an opportunity for human workers to differentiate themselves within the workplace. Several participants noted that human workers, especially those with a disability, can make important contributions to the work environment. Some participants also downplayed the risk of automation to their employment. The fear of machines contributing to job displacement was downplayed in circumstances when a participant was working in a secure job:

I mean, a robot could do a good chunk of my role, but, at the end of the day, I have to gain more strategic leverage to, sort of, overcome that … The people operations are always going to be a factor. I mean, you see bank tellers and the ATM machine coming in, and, yeah, it took some jobs, but now, they phased it, work on client management. I’m not too worried, but I’m not … like, if I’m not in an environment where it’s high stress, high pressure. That is the only thing I worry about …

– Participant ID#146

Overall, the second overarching thematic category highlighted the role of digital technologies as the most prominent driver of change to the nature and availability of work in the future as perceived by young adults with disabilities in our study. Of note, the degree to which a participant described that digital technology would have a positive or negative impact on their employment inclusion was related to whether they held a secure and standard work arrangement. As we note in the following section, digital technology also meant that participants had to reconsider how their job skills could impact their ability to find and sustain employment in the future of work.

### Thematic Category C: barriers and facilitators to obtaining skills to meet the digital transformation of the economy

Stemming from reflections on the digital transformation of the economy, interview participants frequently evaluated the match between their job skills and the types of jobs that could emerge in the future.

#### Theme C1: importance of building technological skills to meet employer demands.

Many participants indicated that having, at minimum, a baseline level of digital literacy would provide a resource that would support workforce entry. One participant who was not working described the need to possess digital literacy, including coding skills, to compete for available entry level jobs in new companies that were increasingly reliant on digital technologies:

A lot of people start as some type of data analyst … A lot of start-ups look for people who just know Python, or even know basic web development, so I had to learn a little bit of HTML and CSS along the way … I do see the utility of having a tangible and hard skill. Because university teaches a lot of soft skills … But, again, it’s not even about the piece of paper [referring to a university degree], it’s about being able to utilize it, like, utilize the skills that they teach you.

– Participant ID#137

Other participants described that the types of digital technologies utilized by employers were changing at a rapid rate, and it was important to continue building more complex technological skills to access career advancement opportunities and meet employer demands.

#### Theme C2: importance of building soft skills to fill gaps in a digital workplace.

Along with the development of technological job skills, participants acknowledged that soft skills (e.g. communication, critical thinking) were valued by employers. Building soft skills could enable young adults to provide uniquely human elements to workplaces that were reliant on digital technology. However, participants noted that the ability to develop soft skills could be limited for young adults with disabilities who face psychosocial barriers in the work environment and may feel socially excluded. For example, one participant in a full-time position at a financial institution described the barriers faced to developing soft skills and the steps to address those barriers.

I’ll take on sort of advisory positions and that’s my way of improving my softer skills, which I think sometimes that’s been also a challenge for people with disabilities. Because often some people with disabilities face extreme social isolation. And that makes it challenging to develop your social skills, which are honestly in the future of work seems to be a bigger thing than an actual degree. I think that’s also a barrier. So, I’m always doing a bit of that as I grow in my career and making sure that I spend time and learning how to articulate my job in effective communication.

– Participant ID#101

#### Theme C3: challenges of obtaining job skills related to disability and work arrangements.

Challenges in obtaining job skills were frequently described by participants and attributed to the demands associated with living with a disability, including the amount of time that was directed towards the management of their disability (e.g. resting, attending healthcare appointments). Other participants indicated that having certain types of disabilities like a mental health or cognitive disability could contribute to limitations to learning new job skills that enabled them to meet changing labor market demands. For example, one participant reflected on how a mental health disability made obtaining a post-secondary education challenging. Based on their previous experience, the participant noted that obtaining new skills to compete for jobs of the future could be difficult:

I finished university a year and a bit ago. And it was very difficult. Originally, when I went into university, I was planning on doing an undergrad and then training to be an occupational therapist. A few years into university, that just had to go, because I could barely finish my BA. And once I was done that, I mean you have no idea the number of all-nighters I pulled to try and get my brain to think the way I needed it to. I was mush by the end of it. So, I couldn’t really think about doing other school.

– Participant ID#164

Relatedly, work arrangements held by a participant at the time of interview frequently determined the extent to which they felt that they could obtain both technological and soft job skills needed for career advancement. Participants employed in contingent work often indicated having a weaker connection to the workplace and felt that their employers were less likely to provide them with job skill development opportunities. Participants working securely were more likely to indicate access to employer-led job skill development opportunities. For instance, one participant who was securely employed in the publishing industry described the provision of training opportunities from their employer so that they could keep up with technological changes within their industry:

Yeah, lately one of the appealing things about this company that I’ve come to was that they give a yearly stipend that you can put towards education. One of the big things in my industry, as much as I talk about how much I love books and the printed books and all this stuff, there’s also been a big movement towards data and data analysis and trend patterns and that kind of thing. I have had it in my head, taken a few classes this last year looking at computer science and statistics, things like that.

– Participant ID#120

Thematic Category C brings to light the importance that young adult participants with disabilities in our research placed on obtaining necessary job skills for the future of work. Similar to Thematic Category B, the ability to gain job skills was often related to whether a participant held a secure work arrangement.

## Discussion

The future of work is rapidly changing all aspects of working life and has important implications for the employment inclusion of young adults with disabilities. Our novel qualitative study examined how the future of work shaped the thoughts and perceptions held by young adults living with disabilities regarding their experiences entering and advancing within the working world. Findings showed that young adults with disabilities prioritize the management of their disability rather than external factors related to the future of work when making career decisions. Above all, the digital transformation of the economy was seen as the most significant aspect of the future of work that could change work contexts and intensify employer requirements for workers with specialized job skills. Furthermore, the work arrangements to which a young adult living with a disability is exposed could contribute to exclusion in the future of work; being employed in contingent work could increase susceptibility to technological disruption and may limit job skills development. Our study advances an understanding of the changing nature of work for young adults with disabilities. Insights can be utilized to inform the design of employment support programming for young adults living with disabilities to promote early and sustained labor market integration.

Traditionally, young adults living with disabilities have faced exclusion from employment opportunities as they have transitioned into the world of work. Our study is one of the first to interview young adults with disabilities on their perspectives regarding different dimensions of the future of work. We found that participants were less likely to think about how different aspects of the future of work could impact long-term employment. Instead, most indicated that their disability shaped their career aspirations and decisions and placed a greater emphasis on the process of finding work and making work-related decisions that prioritized a supportive work environment. Our findings align with past research which highlights the difficulties of transitioning into work with a disability (Jetha et al., 2018). The shorter term perspective articulated by participants in our study may reflect a potential disadvantage for young adults living with disabilities who may be overlooking longer-term exogenous changes within a rapidly changing labor market which have the potential to have more substantial implications for the availability of work. Findings provide preliminary evidence for the design of employment support programming for young adult with disabilities that balances the shorter-term priorities of transitioning into work with a disability with longer-term priorities shaped by forces that characterize the future of work.

In our research, digital technology was recognized as a critical dimension of the future of work that could impact the employment of young adults living with disabilities. Literature on the future of work highlights that the advancement of digital technologies is resulting in large-scale scale changes to the working world, including shifts in the division of labor between human workers and machines (World Economic Forum, 2021; Acemoglu and Restrepo, 2020). For young adults with disabilities, the shift in the division of labor may substantially alter physical and psychosocial job demands, job control and other aspects of the work context in ways that can create barriers and facilitators to employment. For participants in this study, work arrangements shaped perceptions regarding the impact of digital technology on employment opportunities. Those working in contingent employment were more likely to indicate that digital technology would contribute to greater job insecurity when compared to those working in full-time employment. Data consistently indicate that an increasing proportion of jobs are those with contingent and precarious work arrangements (Martin and Lewchuk, 2018; Quinlan et al., 2001). Emerging advancements in AI coupled with accelerated employer investment in digital technologies in response to the COVID-19 pandemic may exacerbate the proportion of precarious jobs (Agrawal et al., 2018, World Economic Forum, 2021, MIT Technology Review Panel, 2020; Kinder and Ross, 2020). The growth in contingent work arrangement coupled with the greater adoption of digital technology may play a role in widen labor market inequities for young adults with disabilities at the early career phase. Overall, our study underscores the vulnerability of precariously employed young adults living with disabilities in the future of work.

On the other hand, securely employed participants in our study described that technology would improve their job performance and increase accessibility when compared to those in contingent work. A recent synthesis of evidence found that digital technologies have the potential to address employment barriers reported by people with disabilities through scheduling flexibility (e.g. virtual telework) and the accommodation of physical limitations (e.g. smart manufacturing robots) or automation of repetitive tasks (e.g. self-driving cars) (Schwab, 2016, Manyika et al., 2017). To build on our findings, research is required to elaborate on the inequities that can emerge from the digital transformation of the economy, including how young adults living with disabilities working in different occupations may be affected the changing nature of work. At the same time, the results from our study highlight the importance of labor market policies and employment programs which pay greater attention to ensuring young adults living with disabilities in contingent employment can obtain pathways to secure sustained employment.

In our study, the future of work was seen by participants as increasing the need to obtain specialized technological and soft skills to obtain jobs of the future and differentiate themselves from machines (Bakhshi et al., 2017; Bick et al., 2020). Yet, participants noted that having a disability as a young person can create unique barriers to accessing skilling opportunities. These challenges could be exacerbated for those in contingent work with limited access to employer-led job skills training. Existing labor market studies project a significant shortage of workers who possesses job skills most required by employers (Miner, 2014; Schneider et al., 2017). For instance, Canadian data estimates that by 2031 there will be a shortage of two million workers who possess specialized technological and soft skills that will be required by employers (Miner, 2014). To address these labor shortages, upskilling and reskilling initiatives targeting all labor market participants have been a major focus of policy and programmatic development (Bick et al., 2020; Moueddene et al., 2019). Our study points to the importance of including persons with disabilities, especially those at the early career phase, in the design of skilling initiatives. Challenges to accessing skilling initiatives has the potential to further exclude persons living with disabilities from higher quality and secure employment in the future of work. Moving forward, research is required to understand how to adapt the design of job skills initiatives for people living with disabilities to promote inclusion in the future of work.

Our study can be contextualized through the lens of biopsychosocial models of disability. Emerging from discussions on the future of work, participants highlighted the importance of the intersection between personal (e.g. educational attainment, possession of job skills), health (e.g. disability characteristics) and changing work context factors (e.g. work arrangements) in creating barriers and facilitators to employment participation. The future of work has the potential to drastically alter the work context to which persons living with disabilities are exposed in ways that contribute to work limitations. Within the context of biopsychosocial models of disability, our research suggests that innovating employment strategies for young adults with disabilities are a priority for a changing work context to promote inclusion in the future of work. Steps at the employer- and policy-levels should also be implemented to ensure that changes to the work and broader labor market contexts do not disproportionately disadvantage young adults with disabilities.

A strength of this study was the inclusion of a diverse group of Canadian young adults with disabilities with a range of work experiences who provided in-depth perspectives regarding their current employment experiences and perceptions regarding the future of work. Also, our research team consisted of multidisciplinary investigative team who provided a unique lens to coding and interpreting emergent themes. Despite an introduction to the future of work concept and probes during the interview process, thinking about work experiences from a future-facing perspective may have been challenging for participants who were focused on the existing impact disability could have on their work. The implications of the future of work may only be truly realized over time as participants are exposed to different technological, sociopolitical, economic and environmental driving forces. We suggest the need for subsequent research to draw on methodologies in the field of future studies and strategic foresight to build scenarios of the future of work that can facilitate insight development and planning to support the inclusive employment of young adults with disabilities (Hines, 2008; Jetha et al., 2022).

## Conclusion

The future of work will shape the experiences of young adults with disabilities as they enter and advance within the working world. Our qualitative study suggests that having a disability limits the extent to which individuals consider various external future of work trends. Indeed, technology is changing the work context to which persons living with disabilities are exposed and job skill requirements. Job security may also play an important role in determining whether a person living with a disability is able to access employment opportunities within the future of work. This is a critical finding because growing precarious working arrangements to which young adults living with disabilities are exposed can contribute to exclusion from a changing labor market. Our research highlights the need for an evolving body of research to determine how emerging trends in the future of work can disrupt the employment of young adults with disabilities.

## Figures and Tables

**Table 1.** Descriptive characteristics of study participants^‡^

Participant ID number^‡^	Age (years)	Gender	Disability type	Employment status and work arrangement	Industry category^‡^
001	31	Female	Physical disability	Full-time Permanent contract	Finance and insurance
002	32	Male	Physical disability	Full-time Permanent contract	Professional, scientific, and technical services
005	25	Female	Mental health or cognitive disability	Full-time Temporary contract	Public administration
017	28	Female	Mental health or cognitive disability	Full-time Permanent contract	Health care and social assistance
021	34	Male	Physical disability	Part-time Temporary contract	Other services
028	24	Female	Mental health or cognitive disability	Unemployed and looking for work	
037	29	Male	Mental health or cognitive disability	Furloughed	
046	29	Male	Mental health or cognitive disability	Full-time Permanent contract	Management of companies and enterprises
047	31	Female	Physical disability	Part-time Temporary contract	Educational services
064	32	Female	Mental health or cognitive disability	Part-time Temporary contract	Other services
069	30	Female	Physical disability	Part-time Temporary contract	Educational services
073	35	Female	Mental health disability	Full-time Temporary contract	Professional, scientific, and technical services
083	32	Female	Physical disability	Furloughed	Other services
088	36	Female	Mental health disability	Part-time Temporary contract	Educational services
093	35	Male	Physical disability	Full-time Permanent contract	Professional, scientific, and technical services
097	29	Female	Physical disability	Full-time Permanent contract	Health care and social assistance
100	25	Male	Physical disability	Part-time Temporary contract	Educational services
117	28	Female	Physical disability	Full-time Permanent contract	Educational services
120	35	Female	Physical disability	Full-time Permanent contract	Arts, entertainment and recreation
164	29	Non-binary	Mental health or cognitive disability	Full-time Permanent contract	Healthcare and social assistance
165	29	Male	Mental health or cognitive disability	Furloughed	Utilities
167	29	Female	Physical disability	Part-time Temporary contract	Finance and insurance

**Note(s):** ‡ = To preserve the privacy of study participants and to align with study research ethics agreement additional sample characteristics could not be presented. Full-time work refers to those working ≥ 30h per week; Parttime work refers to those working < 30h per week; Furloughed workers refer to those temporarily not working at the time of the interview due to the COVID-19 pandemic; ‡ = Industry categories based on standardized Canadian industry codes

**Source(s):** Table by authors
